# A Rare Case of Large-Cell Neuroendocrine Carcinoma Metastasizing to the Brain: A Case Report

**DOI:** 10.7759/cureus.70615

**Published:** 2024-10-01

**Authors:** Yousif Mohamed, Aya Salih, Omer Suliman, Ibrahim Hamad, Ahmed Hussein

**Affiliations:** 1 Radiology, University of Khartoum, Khartoum, SDN; 2 Internal Medicine, University of Khartoum, Khartoum, SDN; 3 Internal Medicine, Harlem Hospital Center, New York, USA; 4 Pathology, El Imam El Mahdi University, Khartoum, SDN; 5 Pathology and Laboratory Medicine, University of Texas at Houston, Houston, USA

**Keywords:** brain metastasis, chromogranin, ct scan, large-cell neuroendocrine carcinoma, neuroendocrine tumors, radiological findings, radiology, synaptophysin

## Abstract

Neuroendocrine tumors (NETs) encompass a diverse spectrum of neoplasms that can originate from various sites, including the gastrointestinal tract. Brain metastases from neuroendocrine tumors, while rare, present significant clinical challenges. In this case report, we present the unique instance of a 50-year-old female with a history of gastrointestinal neuroendocrine tumor who manifested left-sided weakness, tremors, and recurrent focal convulsions. Initial imaging scans revealed a lesion in the right parietal lobe, which was surgically excised and diagnosed as a metastatic large-cell neuroendocrine carcinoma. Post-surgery, the patient's condition stabilized, but she was subsequently advised to chemotherapy. This case underscores the infrequency of brain metastases in the context of gastrointestinal neuroendocrine tumors, underscoring the need for comprehensive screening in such scenarios. Given the aggressive nature of neuroendocrine carcinomas and their propensity to disseminate to the brain, early detection and intervention are crucial. Our rare case also underscores the importance of distinguishing high-grade neuroendocrine carcinomas, which necessitate intensive management, from less aggressive NETs and other metastatic neoplasms that have different treatment approaches.

## Introduction

Neuroendocrine tumors (NETs) are a diverse range of neoplasms that develop from neuroendocrine system cells, which produce and release hormones. While these tumors can appear anywhere in the body, they are most frequently found in the gastrointestinal tract and pancreas [1].

Neuroendocrine carcinomas (NECs) are a specific subtype of these tumors, presenting significant clinical challenges due to their varied manifestations and limited understanding of their underlying biology. Despite these challenges, ongoing research holds promise for substantial advancements in the field [1-3]. Neuroendocrine carcinomas commonly originate in the lungs and gastrointestinal tract [4]. Gastrointestinal neuroendocrine carcinomas (GI-NECs) are a rare and aggressive form of gastrointestinal cancer, constituting 35-55% of all extra-pulmonary neuroendocrine carcinomas [5].

Two distinct histopathological entities within these tumors exist: small-cell neuroendocrine carcinoma and large-cell neuroendocrine carcinoma. These tumors are defined by markers of neuroendocrine differentiation, with synaptophysin and chromogranin A being essential proteins. Synaptophysin, typically found in neurons, is commonly positive in gastrointestinal neuroendocrine carcinomas, while chromogranin A, found in neuroendocrine cells, is less frequently present. Both synaptophysin and chromogranin A indicate a more mature tumor and are considered a favorable prognostic sign [6]. Understanding these markers is crucial for diagnosing, treating, and prognosis of gastrointestinal neuroendocrine carcinomas. The WHO classification system recognizes two distinct families distinguished by genetic, morphology, and clinical behavior: Well-differentiated NETs are defined as neuroendocrine tumors (NET G1, G2, G3), while poorly differentiated ones are described as neuroendocrine carcinoma (NEC, G3) and further subdivided into small and large cell carcinoma. The expression of synaptophysin and chromogranin A, Ki-67, and morphology characterizes all NETs.

In patients with neuroendocrine carcinoma, brain metastases can occur infrequently but may still present with specific symptoms that help in diagnosis and treatment. These symptoms include headaches (seen in over 95% of patients), personality changes and unstable gait (up to 25% of patients), cranial nerve deficits (more than 10% of patients), and seizures (less than 10% of patients). The rarity of brain metastases in gastrointestinal neuroendocrine carcinoma poses a unique challenge for both healthcare providers and patients. It is important to note that around 60-70% of cancer patients with intracranial metastases experience symptoms [7].

Recognizing the significance of precise diagnosis for neuroendocrine carcinomas is crucial. It helps determine the appropriate treatment and management strategies and enables healthcare professionals to make well-informed decisions [3]. Imaging techniques, such as computed tomography (CT) scans, can offer valuable insights into the characteristics of these tumors. Larger tumor size, transmural invasion, circumscribed growth patterns, and areas of cystic change or necrosis are indicative of neuroendocrine carcinomas as opposed to the more benign neuroendocrine tumors [2]. Regarding brain metastasis, magnetic resonance imaging with gadolinium is more adept at detecting these lesions than computed tomography. The location of the metastasis is often influenced by the nearest vascular clusters, with the cerebral hemispheres being the most frequent location, followed by the cerebellum and brain stem [8].

Immunohistochemical analysis of a biopsy or surgical specimens, including staining for synaptophysin and chromogranin A, is also vital [3]. Once a diagnosis of neuroendocrine carcinoma is confirmed, it is crucial to determine the appropriate treatment plan. For localized cases, the primary treatment is surgical resection, often complemented with adjuvant chemotherapy or radiotherapy. In advanced or metastatic cases, systemic therapies like platinum-based chemotherapy, targeted agents, and immunotherapy have shown varying effectiveness [9].

Patients with brain metastases or leptomeningeal carcinomatosis often have poor prognoses and limited treatment options, emphasizing the significance of ongoing research. Engaging in continuous study and discussion is vital for our community [3,9]. To enhance these patients' survival and quality of life, a multimodal approach involving surgery, stereotactic radiosurgery, and whole-brain radiotherapy is necessary [10].

## Case presentation

A 50-year-old female presented with a medical history significant for bowel resection secondary to a gastrointestinal neuroendocrine tumor. Eighteen months later, she presented with a four-month history of left-sided weakness and tremors. She denied any associated sensory changes or headaches. The patient reported recurrent focal convulsions (five episodes) but no loss of consciousness, sphincteric disturbance, or memory deficits. Physical examination revealed decreased muscle tone, diminished power (grade 0), and reflexes in the left extremities, with intact coordination and higher cerebral function. The patient had a history of fibroid resection seven years ago. Laboratory studies indicated normal blood parameters, except for a heightened white blood cell count (32.2 x 10^3^/µL) with a predominance of neutrophils (82%) and low calcium levels of 7.2 mg/dl (Table 1).

**Table 1:** The patient laboratory parameters and reference values

Parameters	Patient values	Reference values
Blood urea	20 mg/dl	15 - 50 mg/dl
Serum creatinine	0.9 mg/dL	0.2 - 1.2 mg/dl
Serum Potassium	3.7 mmol/L	3.5 - 5 mmol/l
Serum Sodium	138	135 - 150 mmol/l
Serum Calcium	7. 2 mg/dL	8.2 - 10.6 mg/dl
WBCs	32.2 x 10^3^/µL	(4 - 11) x 10^3^/µL
Lymphocytes count (%)	2.3 x 10^3^/µL (7.2%)	(2.5 - 10.5) x 10^3^/µL
Neutrophils count (%)	27.1 x 10^3^/µL (84.2%)	(6 - 23.5) x 10^3^/µL
RBCs	4.99 x 10^6^/µL	(3.5 - 5.5) x 10^6^/µL
Hemoglobin	12.8 g/dl	11.6 - 16 g/dl
Platelets	274 x 10^3^/µL	(150 - 450) x 10^3^/µL

**Figure 1:**
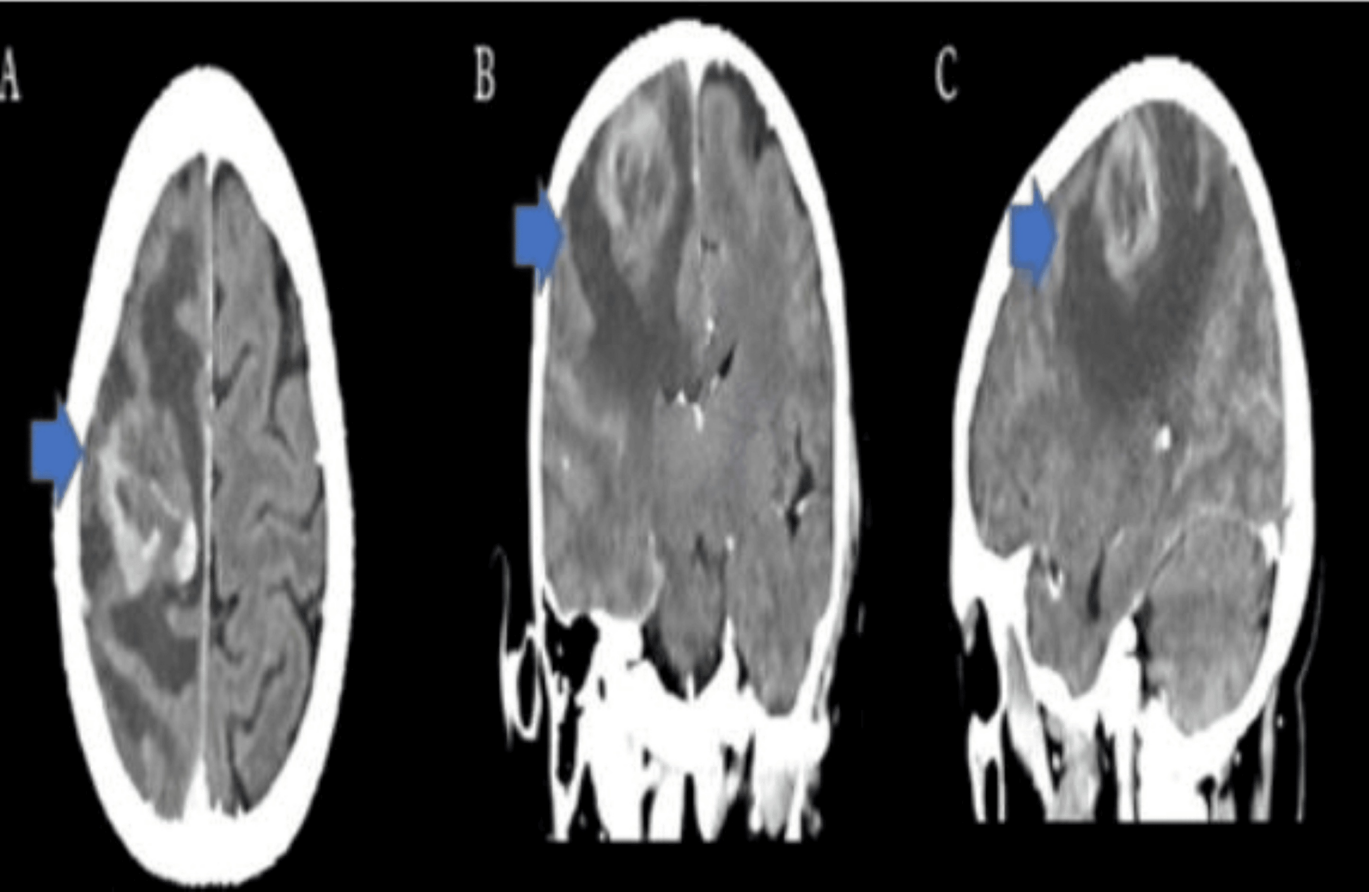
Axial (A), Coronal (B), and Sagittal (C) views of a contrast enhanced CT scan show an ill-defined space-occupying lesion measuring 3 x 3.9 x 3.4 cm in the right parietal lobe, surrounded by marked perifocal hypodense brain edema (highlighted by blue arrows)

**Figure 2:**
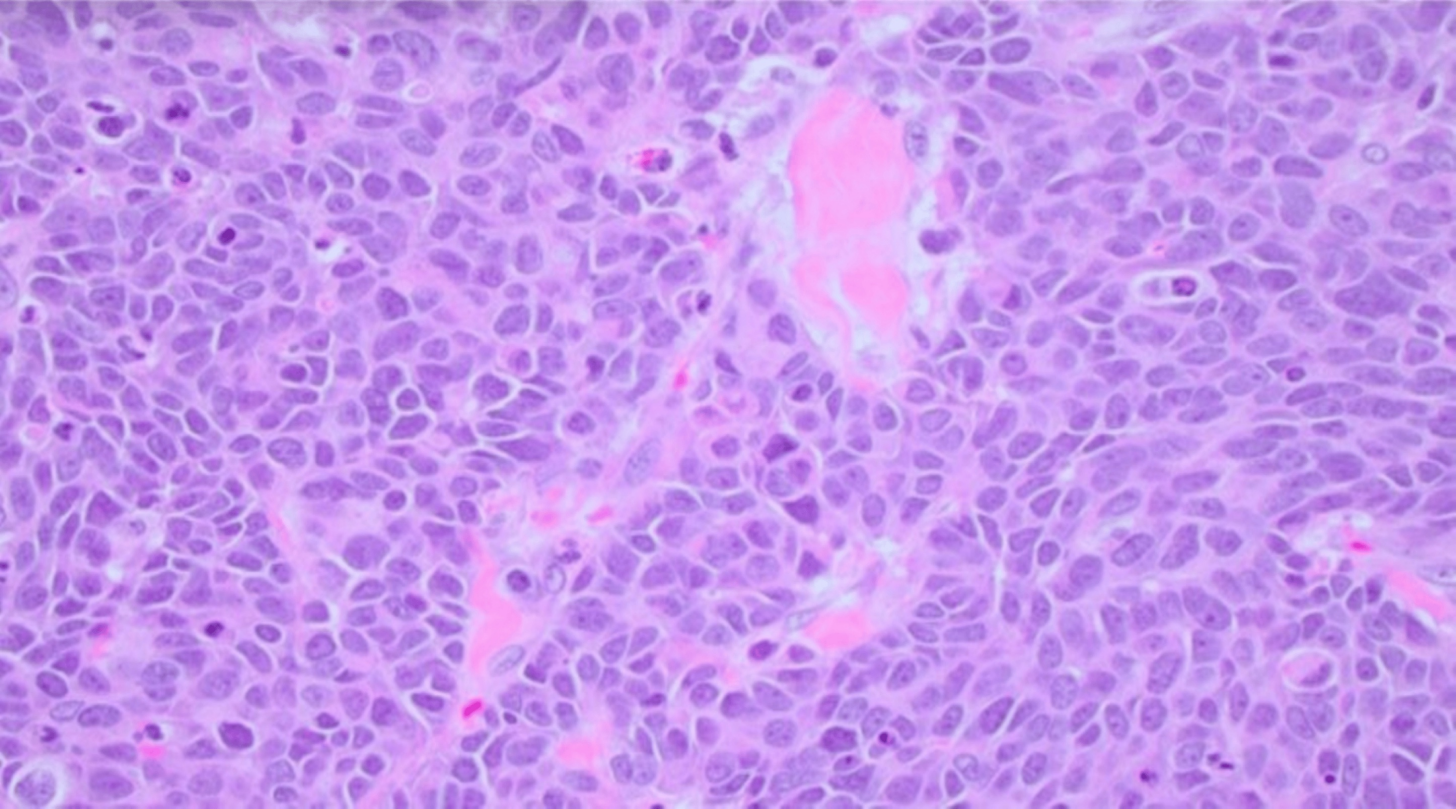
A high-power view (200X) of large-cell metastatic gastrointestinal neuroendocrine carcinoma (GI-NEC) shows cells with enlarged nuclei, prominent nucleoli, and abundant mitotic figures using hematoxylin and eosin (H/E) stain.

**Figure 3:**
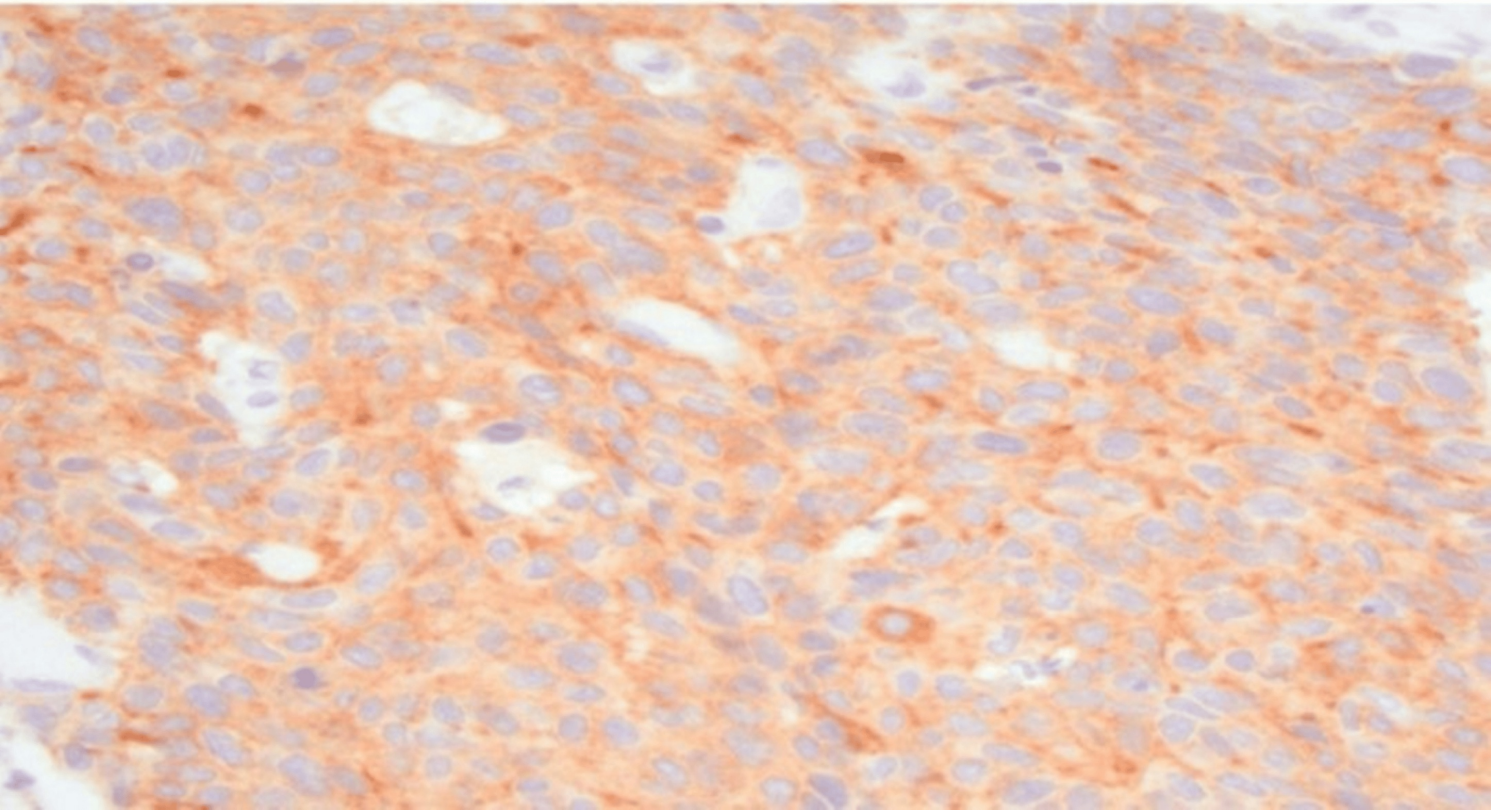
A high-power view (200X) of large-cell metastatic gastrointestinal neuroendocrine carcinoma (GI-NEC) shows that the cells stain positive using synaptophysin stain.

**Figure 4:**
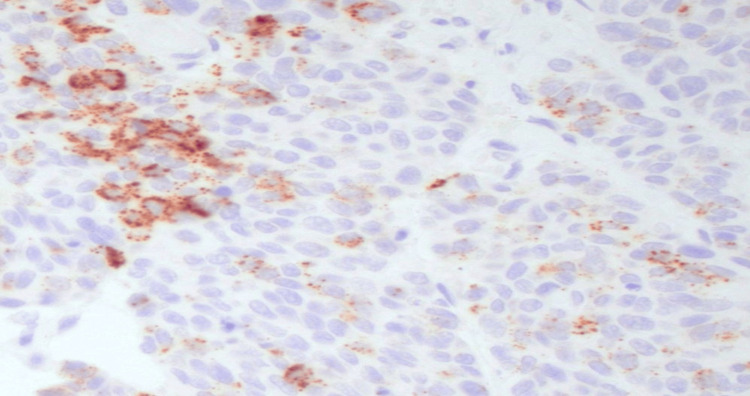
A high-power view (200X) of large-cell metastatic gastrointestinal neuroendocrine carcinoma (GI-NEC) shows that the cells stain positive using chromogranin A stain.

Cerebral computed tomography (CT) scan with contrast, a thorough and meticulous procedure, identified an ill-defined intra-axial space-occupying lesion measuring 3 x 3.9 x 3.4 cm in the right parietal lobe, surrounded with pure vasogenic edema, with no evidence of infarction, intracerebral, or extra-axial hemorrhage, and with normal appearance of the posterior fossa structures (brainstem and cerebellum) (Figure 1). Surgical exploration uncovered an 8 cm firm, irregular intra-axial lesion, which was resected, and a biopsy was obtained for histopathological evaluation; hematoxylin and eosin (H/E) staining revealed features suggestive of a metastatic large-cell neuroendocrine carcinoma (Figure 2), confirmed by synaptophysin (Figure 3) and chromogranin (Figure 4) immunohistochemistry (IHC) stains. Following the procedure, the patient received intensive care unit (ICU) management and remained asymptomatic without deterioration for one week. The patient was transferred to Egypt for further evaluation and treatment due to the challenging circumstances in Sudan.

## Discussion

Neuroendocrine tumors encompass diverse growths that develop from neuroendocrine system cells, which are present in various body parts and responsible for regulating physiological functions. These tumors can emerge in different areas, such as the gastrointestinal tract, pancreas, lung, and central nervous system [11]. In our instance, the primary tumor is located in the gastrointestinal tract, specifically in the jejunum.

Gastrointestinal neuroendocrine tumors, originating from neuroendocrine cells in the digestive system, make up a significant portion of all neuroendocrine tumors, potentially up to 65% of cases [12,13]. Due to the complexity of the condition, diagnosis involves a combination of clinical symptoms, hormone level assessment, imaging, and histological confirmation. Around 50% of patients are diagnosed with metastatic disease. Accurate identification is crucial for guiding treatment and improving patient outcomes [12]. Two main categories of neuroendocrine tumors are well-differentiated neuroendocrine tumors and poorly differentiated neuroendocrine carcinomas. Well-differentiated neuroendocrine tumors are further divided into low-grade and intermediate-grade tumors, while poorly differentiated neuroendocrine carcinomas are considered high-grade.

The metastatic potential differs between neuroendocrine tumors and neuroendocrine carcinomas, with the latter being more likely to develop metastatic disease. For instance, 30.20% of poorly differentiated neuroendocrine carcinoma patients and only 7.12% of well-differentiated neuroendocrine tumor patients develop metastatic disease [14]. Metastatic disease, tumor differentiation, and proliferation rate are important prognostic factors for these neoplasms. Unfortunately, our patient has been diagnosed with neuroendocrine carcinoma with distal metastatic disease to the brain. It is challenging to determine the exact prevalence of brain metastases from these types of tumors due to the rarity of the primary tumors and the lack of large-scale studies [7].

Case reports offer valuable insights, but they do not have the statistical power to establish definitive conclusions. Limited statistics suggest that brain metastasis usually occurs just over a year after diagnosis, often presenting with local recurrence or metastasis to another organ. In our case, the time between the removal of the original neuroendocrine carcinoma and the appearance of metastatic symptoms was approximately fourteen months, with no other apparent metastatic locations. However, additional evaluation and assessment are needed in these cases.

It is essential to be able to differentiate between solitary metastatic neuroendocrine carcinoma and high-grade gliomas as it impacts treatment and patient care. While these two conditions can have similar imaging features, it is crucial to carefully distinguish them based on the characteristics of the surrounding area [15]. High-grade gliomas typically show neoplastic cell infiltration in the surrounding region, while solitary metastatic neuroendocrine carcinoma is associated with pure vasogenic edema. In our patient's computed tomography(CT) scan, an ill-defined lesion in the right parietal lobe was observed with specific characteristics that pointed toward metastatic lesions rather than a high-grade glioma.

It is crucial to utilize histopathology and specific stains to differentiate and confirm the diagnosis of neuroendocrine carcinomas. The metastatic neuroendocrine carcinoma may appear more undifferentiated and anaplastic. Immunohistochemical staining is a valuable tool in diagnosing metastatic neuroendocrine carcinoma, as these tumors often express neuroendocrine markers such as chromogranin, synaptophysin, and CD56, which can aid in distinguishing them from other types of metastatic carcinoma [16].

The histological classification of neuroendocrine carcinomas is based on their cellular characteristics, which can indicate their differentiation patterns. One type of neuroendocrine carcinoma is large cell neuroendocrine carcinoma (LCNEC), which is a rare and aggressive form of neuroendocrine carcinoma sharing features with neuroendocrine-differentiated tumors and specific cytological characteristics, including large cell size, polygonal shape, low nuclear-to-cytoplasmic ratio, high mitotic index, prominent nucleoli, and the presence of necrosis. Differentiating large-cell neuroendocrine carcinoma from adenocarcinoma is crucial due to potential differences in treatment regimens [17]. Another highly aggressive form of neuroendocrine carcinomas is small-cell neuroendocrine carcinoma, characterized by small, round cells with scant cytoplasm and dense chromatin. Additionally, there are less common types of neuroendocrine carcinoma, including atypical carcinoid tumors and well-differentiated neuroendocrine tumors [18]. In our specific case, the histopathology displayed large cell size, a polygonal shape, a low nuclear-to-cytoplasmic ratio, a high mitotic index, prominent nucleoli, and positive synaptophysin and chromogranin immunohistochemistry stains, leading to the diagnosis of large cell neuroendocrine carcinoma.

In managing rare cases of metastatic neuroendocrine carcinoma, the treatment options can vary based on the symptoms caused by brain metastasis and metastatic images [7]. Surgical intervention is primarily palliative, except in cases of poorly differentiated neuroendocrine carcinomas, where chemotherapy is the preferred approach. Surgery remains the method of choice for patients with single brain metastases, with improved outcomes when combined with external beam irradiation. However, the potential benefits of prophylactic brain irradiation in limited-disease neuroendocrine carcinomas of gastroenteropancreatic origin, similar to the approach in small-cell lung cancer, are still not well understood [19].

In individuals with neuroendocrine carcinomas that have spread to the brain, the typical survival time without treatment is around 4-6 months [20]. A study involving 94 patients with gastrointestinal neuroendocrine carcinomas at the National Cancer Registry of Spain revealed a median survival period of just 1.7 months for those with metastatic disease [20]. To improve the prognosis, timely detection and accurate diagnosis play a critical role in prolonging the lives of affected individuals [7].

## Conclusions

The occurrence of brain metastases from gastrointestinal neuroendocrine tumors, though rare, is a possibility, especially in patients with high-grade neuroendocrine carcinomas. Our extraordinary case underscores the critical importance of maintaining a high level of vigilance and closely monitoring neurological symptoms in individuals diagnosed with neuroendocrine tumors. Typically, the management of these metastases involves surgical excision followed by systemic chemotherapy. Early detection is paramount and can significantly influence patient outcomes. Given the infrequency and complexity of these metastases, a comprehensive and multidisciplinary approach, employing the valued expertise of each professional, is imperative for the effective treatment and holistic management of the condition.
